# Pilot-scale co-precipitation synthesis of a novel active ingredient made of ultrasmall iron (oxyhydr)oxide nanoparticles for the treatment of hyperphosphatemia†

**DOI:** 10.1039/d4ra02719a

**Published:** 2024-05-20

**Authors:** Magdalena Teresa Spicher, Sebastian Patrick Schwaminger, Daniela von der Haar-Leistl, Marian Montiel Peralta, Georgina Mikacevic, Friedrich Ernst Wagner, Sonja Berensmeier

**Affiliations:** a Fraunhofer Institute for Process Engineering and Packaging (IVV) Giggenhauser Str. 35 85354 Freising Germany magdalena.spicher@ivv.fraunhofer.de +49 8161 491459; b Chair of Bioseparation Engineering, School of Engineering and Design, Technical University of Munich Boltzmannstraße 15 85748 Garching Germany; c Division of Medicinal Chemistry, Otto Loewi Research Center, Medical University of Graz Neue Stiftingtalstraße 6 8010 Graz Austria; d BioTechMed-Graz Austria; e Department of Physics, Technical University of Munich James-Franck-Straße 1 85748 Garching Germany

## Abstract

Due to its simplicity, co-precipitation is the most commonly used method for producing iron (oxyhydr)oxide nanoparticles. However, it is reported to be sensitive to changes in process parameters, which complicates scale-up and is why only volumes up to 1.2 L have been described in the literature. This study aims to demonstrate the scale-up of a co-precipitation synthesis to 100 L using the example of a new phosphate-binding active ingredient based on iron (oxyhydr)oxide. The synthesis was shown to be very robust to changes in synthesis parameters and stirrer geometries. The *in vitro* phosphate-binding efficacy and the yield were maintained in all five scales tested. Only the content of the components in the nanoparticles varied slightly. However, Mössbauer spectroscopy, dynamic light scattering (DLS), and attenuated total reflection Fourier transform infrared spectroscopy (FT-IR) revealed no evidence of structural changes, but a reduction in the size of the iron (oxyhydr)oxide cores and the total core–shell nanoparticle sizes. Overall, this study has successfully demonstrated that ultrasmall iron (oxyhydr)oxide nanoparticles can be produced on a pilot scale by co-precipitation with a yield of >40 g L^−1^.

## Abstract 2

Ultrasmall iron (oxyhydr)oxide nanoparticles can be prepared with a yield >40 g L^−1^ on a pilot scale by co-precipitation without losing their exceptionally high phosphate-binding efficacy. Only slight changes in composition were observed, attributed to a reduction in core size.

## Graphical abstract

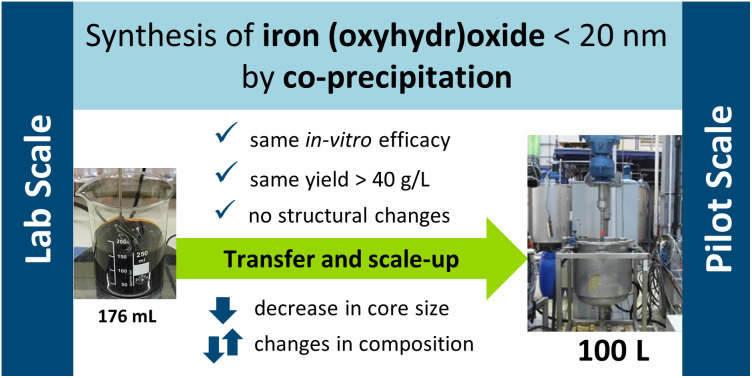

## 1 Introduction

Iron (oxyhydr)oxide nanoparticles are gaining increasing importance in medical applications. As active ingredients, they are inexpensive to produce and are regarded as harmless in terms of their toxicity to living organisms and the environment.^1,2^ In addition, iron (oxyhydr)oxide nanoparticles exhibit good bioavailability and biodegradability and possess high surface/volume ratio and superparamagnetism.^2–4^ Therefore, various promising applications in medicine have been described in recent years, ranging from their use as drug delivery systems,^5,6^ to improving the local treatment of burn wounds,^7,8^ to cancer treatment.^9–11^

Various synthetic methods are known for preparing iron (oxyhydr)oxide nanoparticles, such as microemulsion systems, sol–gel technique, and hydrothermal synthesis.^12^ Due to its simplicity, co-precipitation synthesis is the most commonly used method based on the precipitation of ferric and ferrous ions dissolved in water by adding a base.^5,6,12^ However, it is well-known that numerous influencing factors in co-precipitation synthesis affect the product properties, like the stirring speed, temperature, or the addition time of both reactant solutions.^6,13–15^ On the one hand, this enables the preparation of a wide range of tailored iron (oxyhydr)oxide nanomaterials with various crystal structures and different particle sizes and shapes.^14^ On the other hand, it requires precise synthesis control due to the lower robustness of the process, complicating the scale-up of co-precipitation syntheses and can lead to potential impairment of factors like particle size, product purity, colloidal stability, or surface morphology.^16^ Thus, the synthesis procedures following the co-precipitation route are typically described in research studies with small volumes of up to 1 L and a small amount of product.^11,17–20^ The largest reported co-precipitation process syntheses are in the range up to 1.2 L total volume and 5.4 g L^−1^ of product.^21,22^

To our knowledge, no study has reported a transfer from the laboratory to larger scales and the challenges associated with the scale-up yet. This study aims to demonstrate that ultrasmall iron (oxyhydr)oxide nanoparticles can be produced at a pilot scale-up to 100 L reaction volume despite the sensitivity of co-precipitation synthesis. We perform the scale-up using the example of the production of a newly developed, potentially highly effective active ingredient for the treatment of hyperphosphatemia.^23^ This drug consists of ultrasmall (<20 nm) iron (oxyhydr)oxide nanoparticles enclosed in an organic shell composed of inulin, mannitol, and gum arabic.^24^ The synthesis of this nanoparticle material has been described up to now only for a total volume of 0.2 L with about 8–10 g of product amount.^24^ In this study, we investigate the robustness of the process on a laboratory scale and then transfer the synthesis stepwise up to a reaction volume of 100 L with a production of at least 40 g L^−1^ without limiting the outstanding efficacy of the novel active ingredient.

## 2 Experimental

### 2.1 Materials

We purchased iron(ii) chloride tetrahydrate (FeCl_2_·4H_2_O, ≥99%), iron(iii) chloride hexahydrate (FeCl_3_·6H_2_O, ≥99%), gum arabic (from *Acacia* tree, branched polysaccharide), hydrogen peroxide solution (H_2_O_2_, 30 wt% in H_2_O, puriss), sodium standard solution for ion chromatography (1000 mg L^−1^), d-glucose (≥99%), d-fructose (≥99%), ascorbic acid (C_6_H_8_O_6_, ≥99%), maltitol (C_12_H_24_O_11_, ≥98%), and d-mannitol (C_6_H_14_O_6_, ≥99%) from Sigma-Aldrich. Hach Company produced the chloride test kit (for method 8113), and Berrytec GmbH distributed the 0.2 μm pore size nylon syringe filters. The producer of Inulin HT (food grade) was Spinnrad GmbH, VWR supplied the centrifugal filters (3 kDa, polyethersulfone), and hydroxylamine hydrochloride solution (HONH_2_·HCl, 100 g L^−1^, analytical grade) was obtained from Bernd Kraft. Acetic acid (CH_3_COOH, min 99.8%), ultrapure water (H_2_O, resistivity of 18.2 MΩ cm^−1^, hereafter referred to as water), hydrochloric acid (HCl, min 37.0%), sodium acetate trihydrate (C_2_H_3_NaO_2_·3H_2_O, ≥99.5%), and sulfuric acid (H_2_SO_4_, min 97.0%) were supplied by Chemsolute. We purchased ammonium heptamolybdate tetrahydrate ((NH_4_)_6_Mo_7_O_24_·4H_2_O, analytical grade), Certipur chloride standard (1000 mg L^−1^), sodium dihydrogen phosphate monohydrate (NaH_2_PO_4_·H_2_O, analytical grade), Certipur calcium standard solution (1000 mg L^−1^), Certipur rhodium ICP standard solution (1000 mg L^−1^), iron standard solution (1000 mg L^−1^ in 0.5 M nitric acid) and nitric acid (HNO_3_, 65%, p.a.) from Merck. Sodium azide (NaN_3_, ≥99%) and sodium hydroxide pellets (NaOH, ≥99%) were from Carl Roth. Alfa Aesar supplied 1,10-phenanthroline hydrochloride monohydrate (C_12_H_8_N_2_·HCl·H_2_O, ≥99%) and antimony potassium tartrate trihydrate (K_2_Sb_2_C_8_H_4_O_12_·3H_2_O, 99.0–103.0%). Ammonium fluoride (NH_4_F, analytical grade) was distributed by Fluka.

### 2.2 Preparation of the nanomaterial

**Fig. 1:**
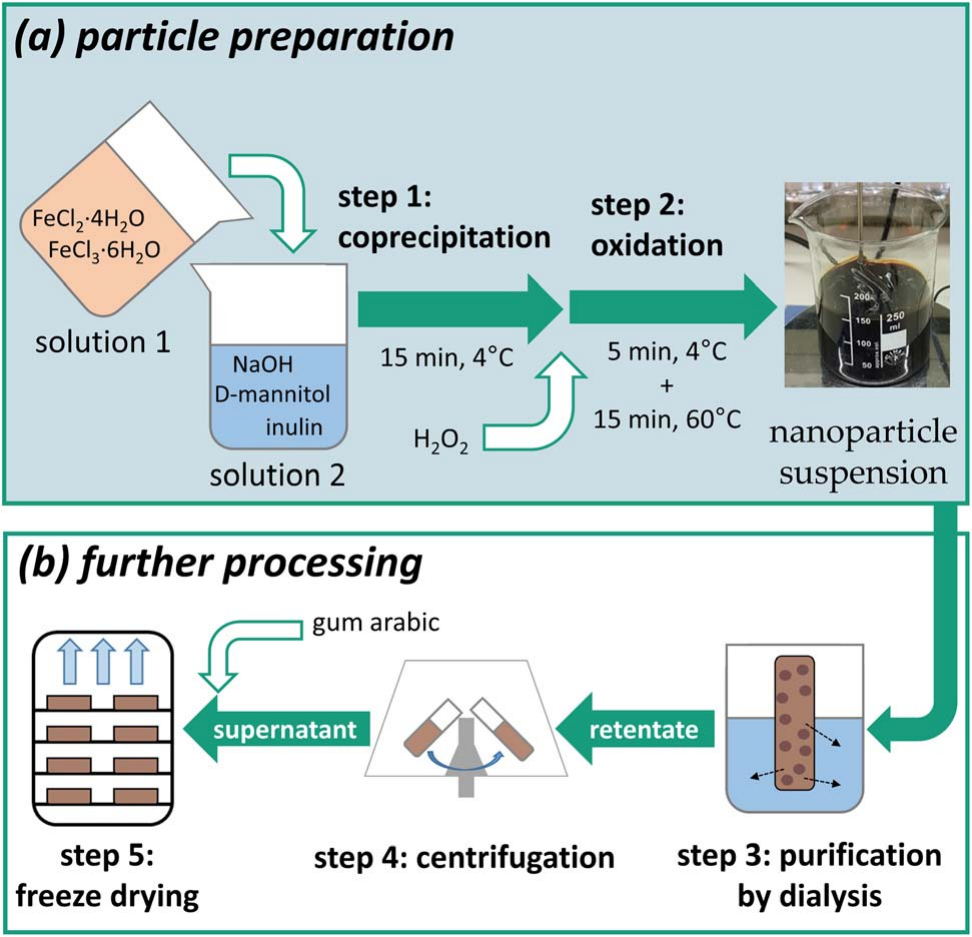
Schematic illustration of the manufacturing process subdivided into (a) particle preparation by co-precipitation and oxidation, and (b) further processing of the nanoparticle suspension.

The iron (oxyhydr)oxide-based nanoparticles were synthesized by co-precipitation according to an adapted protocol reported by Wagner *et al.*^23^ The exact procedure with all further processing steps was reported by Bäumler *et al.*^24^ and is schematically illustrated in Fig. 1.

The production is divided into two sections: (a) particle preparation by co-precipitation synthesis and (b) further processing to refine the particles. This work focuses on section (a). The further steps of section (b) are consistently performed according to the procedure of the laboratory process.^24^ Upscaling of the further steps is part of further studies.^25,26^

#### 2.2.1 Co-precipitation synthesis of the active ingredient nanoparticles in different scales

Two solutions were prepared as initial solutions for the co-precipitation: a solution containing 64 g per L iron(ii) chloride tetrahydrate and 151 g per L iron(iii) chloride hexahydrate in water (solution 1) and another solution consisting of 50 g per L d-mannitol and 150 g per L inulin dissolved in 1.5 M sodium hydroxide (solution 2), both precooled to 4 °C. For the particle preparation (see step 1: co-precipitation in Fig. 1), solution 1 was added as quickly as possible to solution 2 under vigorous stirring. The mixture was stirred at 4 °C for 15 minutes before adding a hydrogen peroxide solution (30%) (step 2: oxidation in Fig. 1) and stirring for additional 5 min at 4 °C. Finally, the stirred suspension was heated to 60 °C for 15 min.

The nanoparticle synthesis was performed in 5 scales from a total reaction volume of 180 mL to 100 L (see Table S1 in the ESI†). The quantity of all solutions used for the preparation can be found for the different scales in Table S1.† The reaction tanks were glass tanks on smaller scales and stainless steel tanks on larger ones (see Table S1†). We use counter-current stirrers with a power input of 18 W m^−3^ (except for 0.2 L, where a propeller stirrer with a power input of 160 W m^−3^ was used). Temperature control was performed using the double-walled reaction vessels with externally connected thermostats. The scale-up was carried out, as far as possible, according to the similarity principle.^27^ From a reaction volume of 1.8 L, the geometric similarity was respected. The agitator geometry and dimensional ratios (ratio agitator diameter/vessel diameter and ground clearance to vessel diameter) were kept constant. In addition, the power consumption per unit volume was kept constant in all scales, as recommended for dispersion processes.^27^ At least two independent syntheses were performed at each scale.

#### 2.2.2 Further processing of the active ingredient nanoparticles

After the synthesis, 180 mL suspension were diluted with 50 mL water and purified by dialysis against water to remove unbound stabilizing molecules and free ions (see step 3: purification by dialysis in Fig. 1). The water was changed three times per day for three days. In step 4, large aggregated particles were removed from the suspension by centrifugation (10 min, 3900*g*) (see Fig. 1). Finally, 1.8 g gum arabic per gram contained iron was dissolved in the suspension before freeze-drying using a Beta 1–8 LMC 2 freeze dryer from Martin Christ Gefriertrocknungsanlagen (see step 5: freeze drying in Fig. 1).

#### 2.2.3 Investigation of the robustness of the synthesis

We investigated the influence of various synthesis parameters at the smallest scale of 0.2 L by a one-factor-at-a-time method. The varied parameters, a brief description, and the respective tested factor levels are shown in Table 1. The standard conditions as described above in section “Co-precipitation synthesis of the active ingredient nanoparticles in different scales” are referred to as level 0.

**Table 1:** Varied parameters for the investigation of the robustness of the synthesis

Synthesis parameter	Description	Level −1	Level 0 (standard conditions)	Level 1	Level 2
Temperature (°C)	Temperature during the co-precipitation (step 1) and the first half of the oxidation (step 2)	—	4	25	40
Power input (kW m^−3^)	Adjusted by the stirring rate during the complete synthesis (steps 1 and 2)	0.03	0.16	0.93	—
Titration time (min)	Addition rate of solution 1 to solution 2 at the beginning of step 1	—	0	6	12
Co-precipitation time (min)	Duration of step 1 (after the addition of solution 1 to solution 2 until the addition of hydrogen peroxide)	10	15	20	30
Oxidation time (min)	Duration of step 2 (after reaching 60 °C for the oxidation)	10	15	20	—

After synthesis, the samples were further processed according to the procedure described above under section “Further processing of the active ingredient nanoparticles”. At least three independent syntheses of each parameter combination were performed.

#### 2.2.4 Testing of different stirrer geometries

We tested different agitators for their suitability for mixing during the co-precipitation at a scale with a total volume of 1.8 L. Table 2 summarizes the selected stirrer types and their main characteristics, such as primary conveying direction and main flow area. All stirrers were self-made of V4A stainless steel (tool number 1.4404). The dimensions (*i.e.*, agitator diameter and distance to the bottom of the vessel) were selected according to the recommendations of Hemming and Wagner^28^ (in the case of the impeller stirrer) and Dialer *et al.*^29^ (for all other stirrers) (see Table 2).

**Table 2:** Agitator types tested in this study and their characteristics^28–30^

Characteristic	Propeller^a^	Impeller	Disk^a^	Pitched-blade	Counter-current
Number of blades	3	3	6	4	2
Stages	1 and 2	1	1 and 2	1	2
Primary conveying direction	Axial	Radial	Radial	Axial, radial	Axial, radial
Recommended viscosity range (Pa s)	<0.5	<0.5	<0.5	<0.5	0.5 to 5
Agitator diameter *d* (cm)	4.0	7.4	4.4	4.3	7.4
Distance to the bottom of the vessel (cm)	4.0	0.9	4.4	3.0	1.1
Stirring speed *n* (min^−1^)	467	150	186	261	158

a Tested as single- and two-stage stirrer.

The stirrer speed was individually adapted for all stirrers to achieve a power input *P*/*V* of 18 W m^−3^ according to eqn (1).1
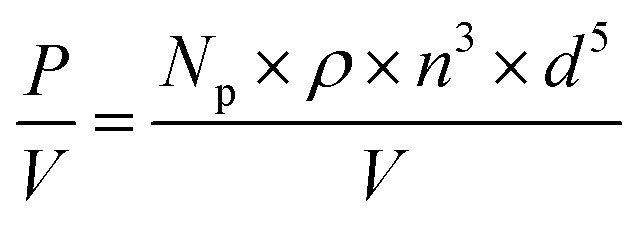
where *N*_p_ is the power number, *ρ* is the fluid density at 4 °C, *n* is stirring speed, *d* is the agitator diameter, and *V* is the synthesis volume. We used literature data for the Newton number of the individual stirrers.^31,32^ For the two-stage versions of the propeller and the disc stirrer, the same agitator speed was used as for the single-stage version. Thus, the power input for these two syntheses will be up to 36 W m^−3^.^31^ Due to the lack of literature data for two-stage designs with the same experimental setup, an exact calculation of the power input was not performed.

### 2.3 Analysis

#### 2.3.1 Synthesis yield

The synthesis yield *Y* was calculated according to eqn (2) as the quotient of the product mass after drying *m*_(product)_ and the synthesis volume *V*.2
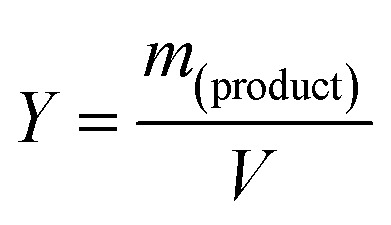


#### 2.3.2 Chemical composition

The composition of the active ingredient was determined according to Spicher *et al.* and Bäumler *et al.*^24,25^ The content of gum arabic *β*(gum arabic) and sodium *β*(Na) was measured by inductively coupled plasma mass spectrometry as previously described by Bäumler *et al.*^24^ We used a 7700 series ICP-MS system (Agilent Technologies) equipped with an ASX-520 autosampler (Agilent Technologies) and a micro mist nebulizer (Agilent Technologies). The system operated with 0.3 rps peristaltic pump speed, 1550 W plasma power, and 15 L min^−1^ flow rate of the argon carrier gas. A solution containing 10 mg per L rhodium in nitric acid (3%) was continuously added during the measurement as an internal standard with a peristaltic pump. 0.12 to 0.24 g active ingredient was dissolved with 10 mL 50% sulfuric acid and diluted to 100 mL with 3% nitric acid. Calcium and sodium standard solutions diluted with 3% nitric acid were used for the calibration. *β*(gum arabic) was calculated indirectly based on the calcium content of the active ingredient.

The content of total iron *β*(Fe) and ferrous content *β*(Fe^2+^) were determined by a 1,10-phenanthroline colorimetric assay. For the quantification of *β*(Fe), 22.5 to 50 mg active ingredient was dissolved with 1 mL hydrochloric acid and diluted to 0.5 L with water. 0.98 mL of a phenanthroline reagent solution (1 g per L 1,10-phenanthroline hydrochloride monohydrate, 14 mL per L acetic acid, and 21.7 g per L sodium acetate trihydrate in water) was mixed with 0.28 mL sample solution and 0.14 mL hydroxylamine hydrochloride solution. For the quantification of *β*(Fe^2+^), 250 to 500 mg active ingredient was dissolved with 1 mL dilute sulfuric acid (50% in water) and 1 mL ammonium fluoride solution (5 mol L^−1^ in water) before diluting the sample solution with water to 50 mL. For the analysis, 0.9 mL of the phenanthroline reagent solution (composition see above) and 0.1 mL of the ammonium fluoride solution was mixed with 0.1 mL sample solution. We measured the absorbance of the sample solutions at 510 nm with a BioTek Synergy HTX Microplate Reader and calculated *β*(Fe) and *β*(Fe^2+^) using calibration curves obtained with iron reference solutions. We used solutions of iron standard diluted in water (for the quantification of *β*(Fe)) and diluted in 240 mL per L hydroxylamine hydrochloride solution (for the quantification of *β*(Fe^2+^)). The share of ferrous ions *c*(Fe^2+^) was calculated as follows:3
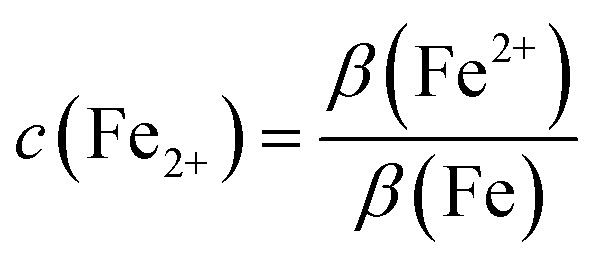


We used high-performance anion exchange chromatography, as previously described by Spicher *et al.*,^4^ to quantify mannitol and inulin. The Dionex ICS-3000 Ion Chromatography System was equipped with a pulsed amperometric detector, a CarboPac PA10 column (Thermo Fisher Scientific) and a guard column (Thermo Fisher Scientific). Eluent A was water, eluent B 150 mM NaOH and eluent C 150 mM NaOH with 1 M sodium acetate. The flow rate was 0.25 mL min^−1^ with a temperature of 25 °C. For the measurements, the eluents were mixed as follows: 0–25 min: 90% A + 10% B; 30–35 min: 100% C; 40–55 min: 90% A + 10% B. 50 mg active ingredient was mixed with 1.5 mL 2% hydrochloric acid and heated to 80 °C for 60 minutes. 30 μL of this solution was diluted with 0.1 M sodium hydroxide to 10 mL and 500 μL was mixed with 1.5 mL internal standard solution (10 g per L sodium azide and 0.2 g per L maltitol in water). Before analysis, all sample solutions were filtered with a 0.2 μm nylon syringe filter. The content of mannitol *β*(Man) and inulin *β*(Inu) was determined using calibration curves measured with standard solutions containing glucose, fructose, and mannitol. *β*(Inu) was calculated as the sum of the contained glucose and fructose amount.

The chloride content *β*(Cl) was determined using a mercuric thiocyanate photometric assay using a chloride test kit (Hach Company), according to Spicher *et al.*^25^ We dissolved 30 mg of the active ingredient with 2 mL 3% nitric acid within 10 min at 80 °C. For the quantification, we used a standard addition method: we added defined amounts of 0 to 3 μL chloride standard solution (1000 mg L^−1^) to the sample solutions and diluted the mixtures to 1 mL with water. For the spectroscopic measurement, 160 μL mercury thiocyanate solution and 80 μL ferric ion solution, both contained in the test kit, were added to the samples, and the absorbance at 455 nm was measured with a BioTek Synergy HTX Microplate Reader. *β*(Cl) was calculated by linear regression.

The residual moisture RM was determined by Karl Fischer titration using a Karl Fischer KF titrator Aqua 40.00 (ECH Elektrochemie Halle GmbH) equipped with a headspace module. 10 to 20 mg of the active ingredient was filled in a headspace vial, sealed with an aluminum cap, and heated in the headspace module to 90 °C. The evaporation moisture was quantified until the following drift stabilization was achieved: increase ≤ initial drift + 2 μg min^−1^.

#### 2.3.3 *In vitro* phosphate-binding efficacy

The phosphate-binding efficacy of the active ingredient was determined according to Spicher *et al.*^26^ The *in vitro* test conditions were selected based on the average residence time of about 2 hours and the pH environment in the stomach of pH 3 and in the small intestine of pH 7.^33^ 350 mg active ingredient was dispersed in 5 mL water and 5 mL phosphate solution (22.77 g per L sodium dihydrogen phosphate in water), adjusted with 1 mol per L hydrochloric acid and 1 mol per L sodium hydroxide solution to pH 3 and pH 7. The sample solution was incubated for 2 hours at 41 °C at 500 rpm in an Eppendorf ThermoMixer, and the pH was adjusted regularly. Afterwards, the supernatant was received with a centrifugal filter at 12 000*g* for 10 min. The nanoparticles were removed from the samples after two hours using a centrifugal filter at 12 000*g* for 10 min, and we determined the concentration of the unbound phosphate in the filtrate *c*_filtrate_(PO_4_^3−^), as well as the iron concentration *c*_dispersion_(Fe) and the phosphate concentration *c*_dispersion_(PO_4_^3−^) in the dispersion. *c*_dispersion_(Fe) and the iron content in the active ingredient *β*(Fe) were measured as described above. Phosphate was quantified using a molybdate photometric assay according to DIN EN ISO 6878.20.^34^ The phosphate-binding efficacy was calculated according to eqn (4):4
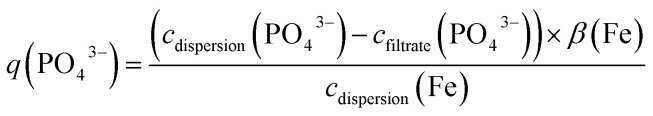


In addition, we calculated the volumetric binding capability *M*(PO_4_^3−^), which correspond to total mass of phosphate that could be bound with the active ingredient produced per L synthesis volume, according to eqn (5). *M*(PO_4_^3−^) enables to compare the effect of a different synthesis yield and binding efficacy (*e.g.* for an active ingredient sample with a low synthesis yield but with a high phosphate-binding efficacy).5
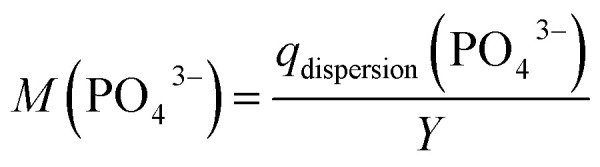


#### 2.3.4 Attenuated total reflection Fourier transform infrared spectroscopy

We used a Bruker ALPHA II FT-IR spectrometer with a platinum attenuated total reflection module for attenuated total reflection infrared Fourier transform spectroscopy (FT-IR). 20 mg of freeze-dried nanoparticles were dispersed in 20 mL of water and ultrasonicated for 10 min. The sample dispersions were dropped on the crystal, and the water was removed with a dryer. We determined 24 scans per spectrum and subsequently corrected the baseline with a “rubber band” method with the OPUS software. We normalized each spectrum to the most intensive peak in the range of 4000 to 450 cm^−1^.

#### 2.3.5 Mössbauer spectroscopy

Mössbauer spectroscopy was measured according to Bäumler *et al.*^24^ in transmission geometry with a standard electromechanical spectrometer (Halder Elektronik) using a source of ^57^Co in rhodium and a sinusoidal velocity waveform. Calibration was performed with an α-iron foil at room temperature. The spectra were measured at 300 K and 4.2 K (in this case the source and the absorber were cooled in a liquid He bath cryostat). The spectra were least-square fitted with Gaussian distributions of quadrupole splitting for 300 K spectra and hyperfine fields for 4.2 K-spectra. The data evaluation was carried out as described by Bäumler *et al.*^24^

#### 2.3.6 Dynamic light scattering

A Zetasizer Ultra (Malvern Panalytical GmbH) was used for determining the particle size distribution by dynamic light scattering (DLS). For sample preparation, 20 mg nanoparticles were dispersed in 20 mL purified water and treated with ultrasonic for 10 min.

#### 2.3.7 Transmission electron microscopy

The TEM images were recorded with a JEM-1400(PLUS) 120 kV transmission electron microscope (JEOL GmbH). The nanoparticles were redispersed in a few drops of purified water for sample preparation and dropped onto a carbon-coated copper grid.

#### 2.3.8 Statistical analysis

Each measurement was performed in at least triplicate. Statistical analysis was performed using OriginPro 2022b. It was pretested for homogeneity of variance using Levene's test (*p* < 0.05). If homogeneity of variance was assumed, a one-way ANOVA was performed, followed by a Tukey HSD *post hoc* test (null hypothesis: both results are not significantly different, *p* < 0.05). In the case of heteroscedasticity, a Kruskal–Wallis test, followed by a Dunn's *post hoc* test, was performed instead, with the same hypotheses as for ANOVA.

## 3 Results and discussion

### 3.1 Investigation of the robustness of the co-precipitation synthesis

In general, co-precipitation syntheses of iron (oxyhydr)oxide nanoparticles are known for their sensitivity to changes in particle properties, like particle size, product purity, colloidal stability, or surface morphology, due to variations in synthesis parameters.^6,14,15,35^ In preparation for the scale-up, we initially investigated the sensitivity (or robustness) of this synthesis protocol for this special active ingredient. For this purpose, we varied the synthesis parameters temperature, power input by stirring, duration of the co-precipitation and of the oxidation step, as well as the addition rate of the iron salt solution to the alkaline solution. We investigated the effects on the yield and the *in vitro* phosphate-binding efficacy. Additionally, we determined the particle composition. Due to the limited product quantity, we had to waive chloride quantification. With less than 1%, this impurity represents the component with the lowest proportion.^24^ Due to this small share in the active ingredient and as it is not likely that chloride is toxic in these small amounts, we consider the chloride quantification in the context of the analysis of the synthesis robustness to be dispensable.

The results can be found in Tables S2–S6 in the ESI.† The most critical characteristics of the process are the product yield and the *in vitro* efficacy (as well as the volumetric binding capability). No significant differences are observed for these characteristics when the synthesis parameters are varied. The mean values of all samples are shown in Table 3.

**Table 3:** Average achieved synthesis yield *Y*, phosphate-binding efficacy *q*(PO_4_^3−^), and volumetric binding capability *M*(PO_4_^3−^) for all samples produced to investigate the synthesis robustness (*n* ≥ 39)

Characteristics	Average
Synthesis yield *Y* (g L^−1^)	47.4 ± 3.0
Phosphate-binding efficacy *q*(PO_4_^3−^)_pH 3_ (mg g^−1^)	200.0 ± 12.2
Phosphate-binding efficacy *q*(PO_4_^3−^)_pH 7_ (mg g^−1^)	122.2 ± 12.1
Volumetric binding capability *M*(PO_4_^3−^)_pH 3_ (g L^−1^)	9.4 ± 0.8
Volumetric binding capability *M*(PO_4_^3−^)_pH 7_ (g L^−1^)	5.8 ± 0.7

Regarding the composition, some minor changes in the iron and inulin content can be recognized. The inulin content rises with increasing synthesis temperature, power input, and titration time (see Tables S2–S4 in the ESI†). The iron content, on the other hand, decreases with increasing power input and longer co-precipitation time (see Tables S3–S5 in the ESI†). However, comparable research studies typically only consider the effects on the particle size distribution and the formed crystal structures^6,14,15,35^ but not on the composition of the nanoparticles. We suspect that the observed effects could also be due to variations in the primary particle size or shape. The iron ions are expected to be bound in an iron (oxyhydr)oxide structure in the core of the nanoparticles.^24^ The organic components surround this core as a shell, protecting the nuclei from particle aggregation. A change in the ferrihydrite core size leads to a different volume/surface area ratio, whereby the quantity ratio of iron and organic components can vary. Due to the above-mentioned limited sample quantity, which already forced us to forego a complete analysis of the composition, we could not additionally perform a size determination of the ferrihydrite cores.

**Fig. 2:**
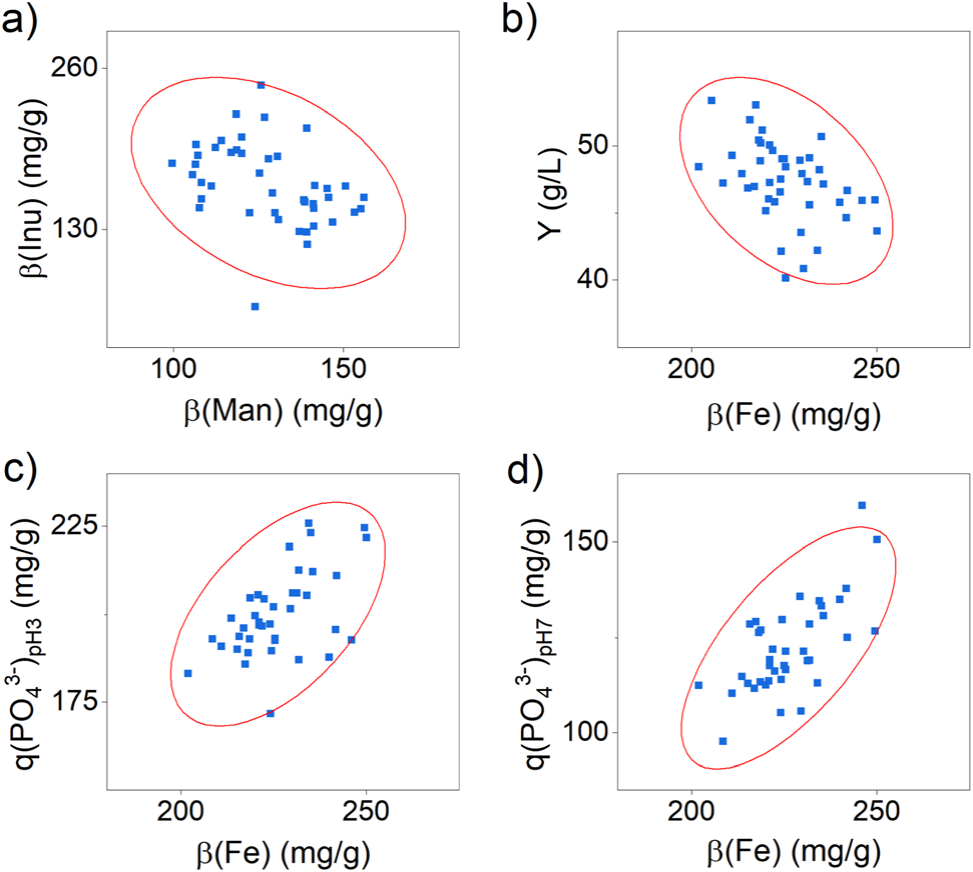
Scatter plots for the correlation between (a) the content of mannitol *β*(Man) and inulin *β*(Inu), (b) the iron content *β*(Fe) and the product yield, (c) the iron content *β*(Fe) and the phosphate-binding efficacy at pH 3 *q*(PO_4_^3−^)_pH 3_ and (d) the iron content *β*(Fe) and the phosphate-binding efficacy at pH 7 *q*(PO_4_^3−^)_pH 7_. The ellipses shown represent the confidence ellipse (calculated as part of the Pearson correlation test *via* Origin Pro 2022b).

Furthermore, it still needs to be clarified in which range the composition of the active ingredient may vary to achieve a constant yield and phosphate-binding efficacy. Since a comparable yield and phosphate-binding efficacy were observed in this test series, we estimate a relatively small effect of the composition. To verify this, we determined possible correlations between the content of the components and the efficacy and yield using statistical correlation analyses. The results are shown in detail in Table S7 and Fig. S1 in the ESI.† Significant correlations were identified between the content of inulin and mannitol (see Fig. 2a) and between the iron content and the product yield (see Fig. 2b), as well as the phosphate-binding efficacy (see Fig. 2c and d).

The results indicate that active ingredient samples with a higher mannitol content have a significantly lower inulin content. Both organic substances are expected to bind to the surface of the iron (oxyhydr)oxide core *via* ligand exchange reactions and electrostatic attraction forces.^24^ They thus compete for the same binding sites, which is why a negative correlation in the content of these two molecules can be observed.

Furthermore, the data demonstrate that a higher iron content leads to a lower yield and, at the same time, to a higher phosphate-binding efficacy (see Fig. 2b–d). Regarding the reduced yield, we assume that particles with a higher iron content are less stabilized and thus tend towards particle growth and aggregation.^36^ As a result, a bigger fraction of particle aggregates is separated in centrifugation in this manufacturing process, which is why the product yield is reduced. The positive influence of the iron content on the phosphate-binding efficacy can be explained by the binding mechanisms of phosphate to iron (oxyhydr)oxides. In general, two possible mechanisms are distinguished: phosphate ions can bind to surface iron ions *via* adsorption and can cause iron ions to dissolve out of the iron (oxyhydr)oxide structure and precipitate to iron phosphate.^37–41^ For the active ingredient of this study, Spicher *et al.* recently reported evidence of both binding mechanisms in phosphate-binding.^4^ In samples containing more iron, more binding sites are available to the phosphate, so that a higher phosphate-binding efficacy can be observed. However, the results of this study also showed that the iron content alone is not decisive for the exceptionally high efficacy, but that the organic components in the active substance also seem to play a decisive role.^4^ But the authors could not finally clarify this connection in their study. The reported results support our observations that, despite minor changes in composition, all samples produced showed comparable efficacy.

Considering these results in the synthesis scale-up, it is particularly worth mentioning that the iron content has an opposite effect on the target parameters yield and phosphate-binding efficacy. While an increasing iron content leads to an increase in efficacy the amount of product decreases simultaneously. It is not possible to make a general conclusion whether a higher yield with a lower efficacy or a lower yield is economically preferable. For this reason, we additionally calculated the volumetric phosphate-binding capability *M*(PO_4_^3−^), which indicates the total amount of phosphate the active ingredient per L synthesis can bind. It combines the synthesis yield and the phosphate-binding efficacy in one factor. The correlation analysis indicates no dependence of *M*(PO_4_^3−^) with iron content (see Table S7 in the ESI†).

In summary, we found no significant changes in yield and phosphate-binding efficacy by changing the synthesis parameters. Only slight differences in the inulin and mannitol content were observed. Subsequent correlation analyses indicated that only the iron content affected the yield and the phosphate-binding efficacy. However, particle composition does not significantly influence the volumetric phosphate-binding capability. For this reason, the synthesis is overall very robust and not very sensitive to changes.

### 3.2 Suitability of different stirrer geometries for co-precipitation synthesis

In the initially described procedure, the active ingredient was prepared in the laboratory using a magnetic stirrer.^23^ However, it is essential to use direct-drive mechanisms with a motor for stirring on larger scales due to the limited power provided by a magnetic stirrer.

Based on the general characteristics of the most common agitator geometries for liquid media, we have made a preselection whereby the recommended viscosity range was decisive. Between 4 °C and 60 °C, the synthesis suspension containing the iron (oxyhydr)oxide nanoparticles has a dynamic viscosity lower than 10 mPa s (see Fig. S2 in the ESI†). For this reason, we excluded stirrer geometries that are more suitable for more viscous media, such as the anchor stirrer.^28^ We selected five different stirrer designs (propeller, impeller, disk, pitched blade, and counter-current type) to empirically test their suitability for mixing during the co-precipitation synthesis. In addition, we tested the propeller and the disk agitator as one- and two-stage stirrers.

The active ingredients produced with the different stirrers are all similar in their properties (see Table S8 in ESI†). No significant differences are observable in the composition, the phosphate-binding efficacy, and the synthesis yield, and the parameters are consistent with the observed results of the robustness study (see Table 4).

**Table 4:** Average achieved synthesis yield *Y*, phosphate-binding efficacy *q*(PO_4_^3−^), and volumetric binding capability *M*(PO_4_^3−^) for all samples produced to investigate the suitable stirring geometries (*n* = 14)

Characteristics	Average
Synthesis yield *Y* (g L^−1^)	43.0 ± 4.3
Phosphate-binding efficacy *q*(PO_4_^3−^)_pH 3_ (mg g^−1^)	204.7 ± 10.6
Phosphate-binding efficacy *q*(PO_4_^3−^)_pH 7_ (mg g^−1^)	110.0 ± 9.6
Volumetric binding capability *M*(PO_4_^3−^)_pH 3_ (g L^−1^)	8.8 ± 1.1
Volumetric binding capability *M*(PO_4_^3−^)_pH 7_ (g L^−1^)	4.8 ± 0.8

In summary, all tested agitator types are equally suitable for producing the active ingredient on larger scales. For the further scale-up, we selected the counter-current stirrer.

### 3.3 Scale-up of the co-precipitation synthesis

**Fig. 3:**
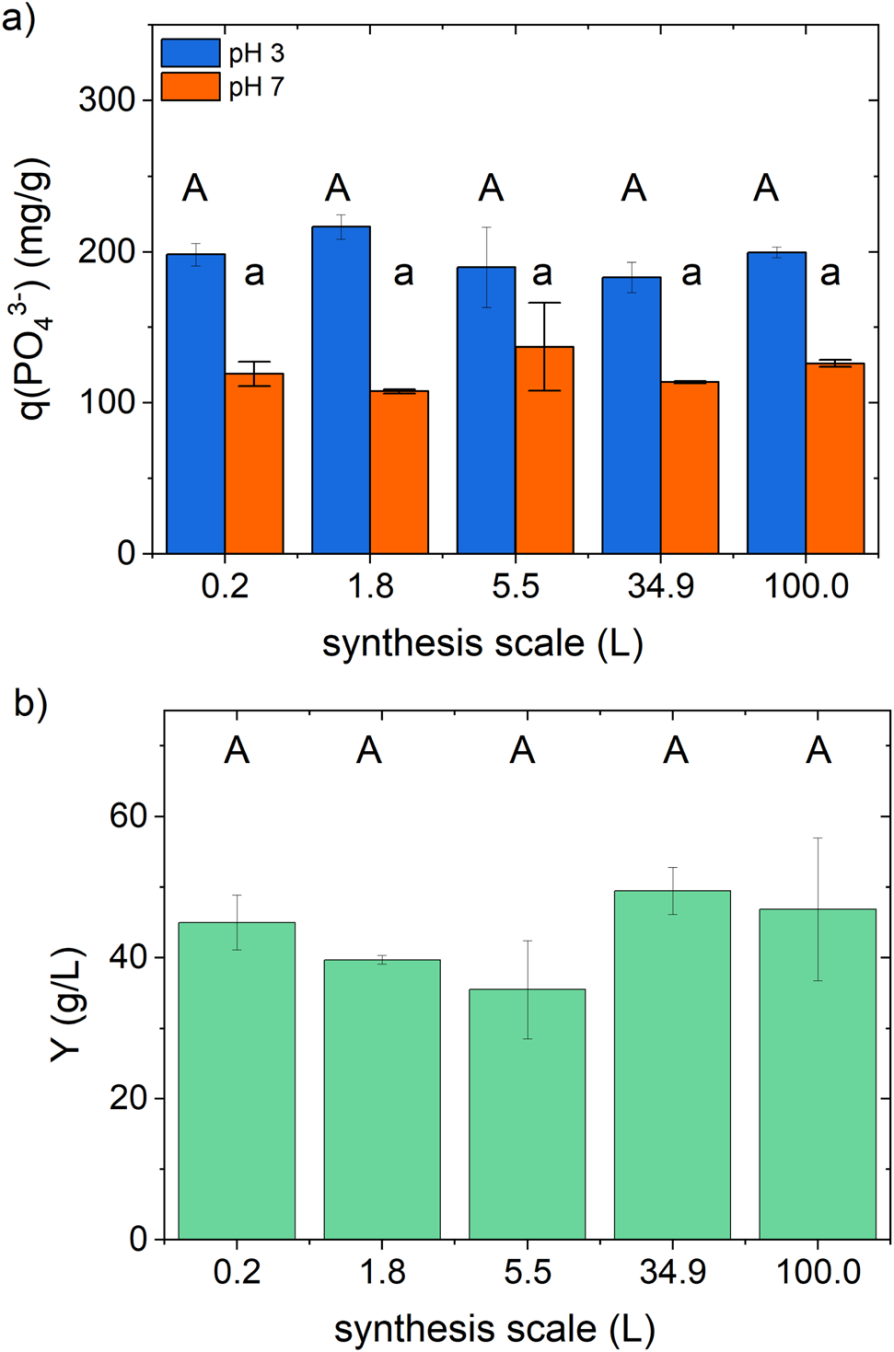
Scale-up of the co-precipitation synthesis: (a) phosphate-binding efficacy *q*(PO_4_^3−^) at pH 3 and 7, and (b) synthesis yield *Y*. The results are the mean value of at least two independent samples prepared under the same conditions on the same scale. Different letters indicate statistically significant differences (*p*-value < 0.05).

We performed the scale-up in five steps from 176 mL to 100 L, corresponding to a final scale-up factor of 568 (see Table S1 in the ESI†). The active ingredient samples prepared in the different scales show a comparable *in vitro* phosphate-binding efficacy and product yield (see graphical illustration in Fig. 3 with the statistical results in Table S9 in the ESI†).

The mean synthesis yield, the phosphate-binding efficacy, and the volumetric binding capability are in good agreement with the observed results of the preliminary studies reported above (see Table 5).

**Table 5:** Average achieved synthesis yield *Y*, phosphate-binding efficacy *q*(PO_4_^3−^), and volumetric binding capability *M*(PO_4_^3−^) for all samples produced for the scale-up of the synthesis (*n* ≥ 10)

Characteristics	Average
Synthesis yield *Y* (g L^−1^)	43.7 ± 6.5
Phosphate-binding efficacy *q*(PO_4_^3−^)_pH 3_ (mg g^−1^)	196.8 ± 17.2
Phosphate-binding efficacy *q*(PO_4_^3−^)_pH 7_ (mg g^−1^)	122.3 ± 17.5
Volumetric binding capability *M*(PO_4_^3−^)_pH 3_ (g L^−1^)	8.5 ± 1.4
Volumetric binding capability *M*(PO_4_^3−^)_pH 7_ (g L^−1^)	5.3 ± 1.1

We also examined the composition of the active ingredient samples, like in the preliminary tests (see Table S9 in the ESI†). Significant differences in the inulin, mannitol, and iron content were found, changing with the synthesis scale, graphically illustrated in Fig. S3 in the ESI.† A systematic increase can be noticed with increasing synthesis scale for the inulin and the mannitol content. Whereas on the laboratory scale, only 120 ± 10 mg per g mannitol and 136 ± 8 mg per g inulin were contained in the active ingredient samples, the content of both substances increased up to 162 ± 4 mg per g mannitol and 228 ± 24 mg per g inulin after synthesis with 100 L total volume. At the same time, a slight reduction in the iron content from 216 ± 4 mg g^−1^ in the laboratory scale to 194 ± 6 mg g^−1^ in the 100 L-synthesis can be observed.

**Fig. 4:**
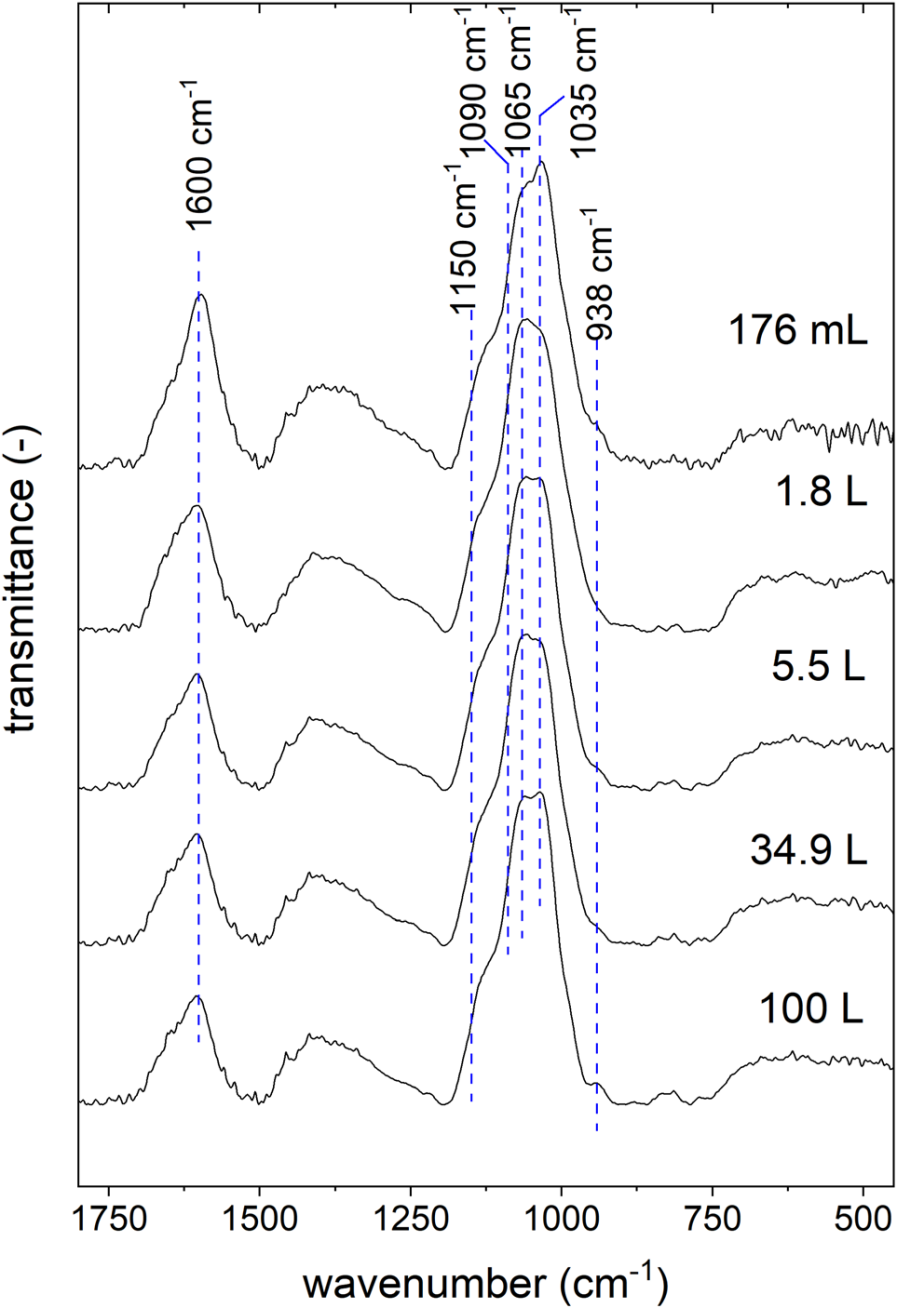
Fourier transform infrared (FT-IR) spectra of active ingredient samples synthesized in different scales.

To exclude changes in structure, we recorded FT-IR spectra of the active ingredient samples of all synthesis scales (see Fig. 4).

There are only slight differences observable in the spectra. The samples produced on the larger scales show a higher peak at 938 cm^−1^, and the shoulder between 1150 and 1090 cm^−1^ is more prominent. These changes can be attributed to the increased inulin content in the active ingredient samples produced on larger scales.^25^ Furthermore, the peaks at 1065 and 1035 cm^−1^ change with the synthesis scale. In this wavelength range, mannitol, inulin, and gum arabic show peaks caused by C–O–C stretching.^42–44^ As recently reported, different content ratios of these three organic components lead to altered shapes of these two peaks.^25^ Lastly, the peak at 1600 cm^−1^ decreases with increasing synthesis scale. This peak is a typical vibration of molecular water in ferrihydrite,^45^ the most probable crystal structure of this active ingredient.^24^ Thus, the spectra indicate a decrease in the ferrihydrite content with the synthesis scale, which is consistent with the observation of the reduction of the iron content and the increase of the organic components. It is also conceivable that the ferrihydrite content is reduced by forming other iron (oxyhydr)oxide structures. Usually, X-ray diffraction (XRD) is applied to study the crystal structure in this type of nanomaterial.^46^

**Fig. 5:**
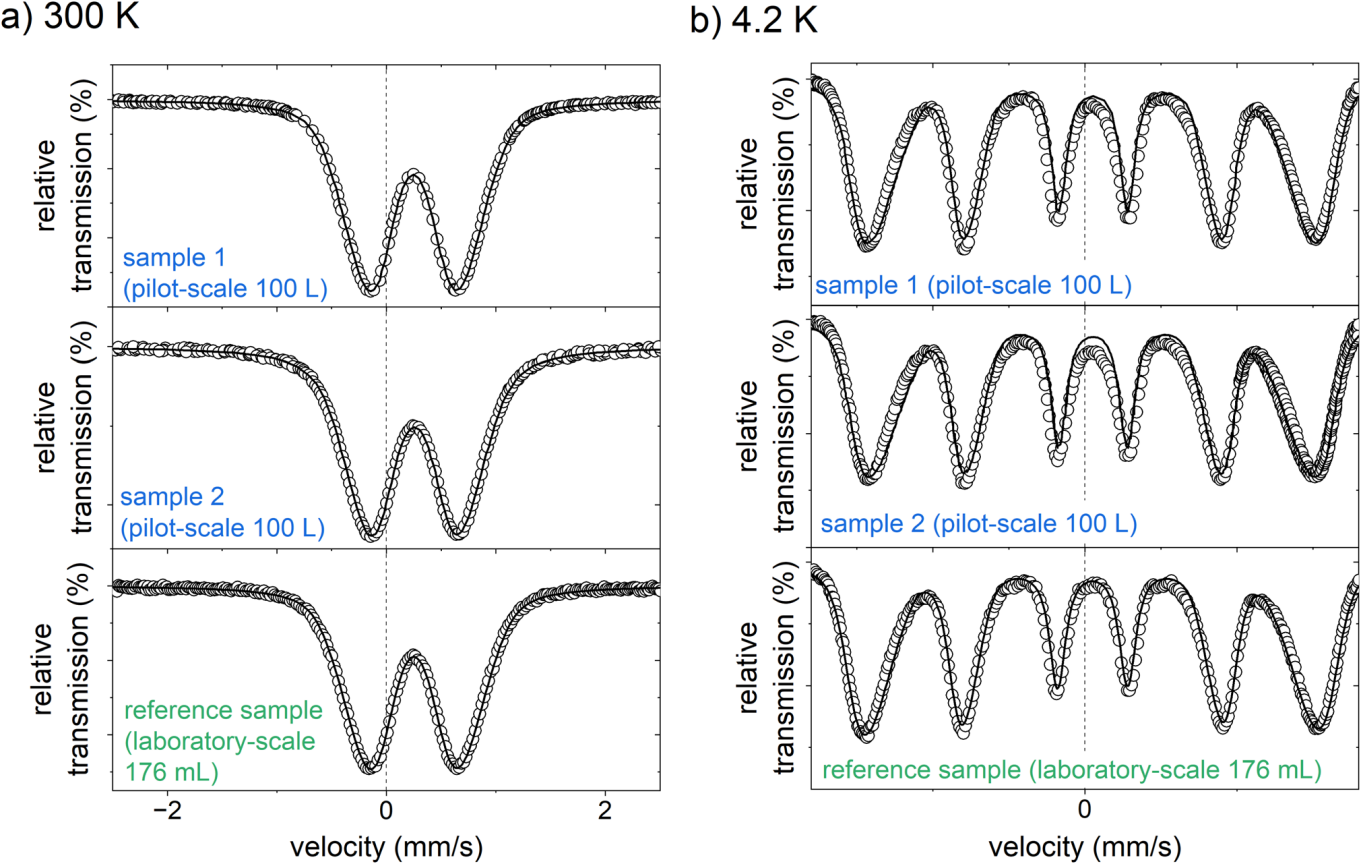
Mössbauer spectra of the two active ingredient samples prepared on a pilot-scale (100 L), and the reference sample, produced on a laboratory scale (176 mL, data reproduced by Bäumler *et al.*^24^) measured at (a) 300 K, and (b) 4.2 K.

However, it has already been shown in a preliminary study that XRD analyses are challenging for this active ingredient due to the ultrasmall particle size and the low crystallinity.^24^ Instead, Bäumler *et al.* applied Mössbauer spectroscopy, among other methods, to further characterize the iron (oxyhydr)oxide core of this compound.^24^ To obtain information on the environment of the iron ions and thus derive conclusions on potential changes in the crystal structure, we also investigated the active ingredient samples produced on the 100 L scale with this method (see Fig. 5). For comparison, we use the results presented by Bäumler *et al.* on the characterization of this active substance.^24^ The samples analyzed in that study were prepared on a laboratory scale with a synthesis volume of 176 mL according to the procedure described above in section “preparation of the nanomaterial”.

**Fig. 6:**
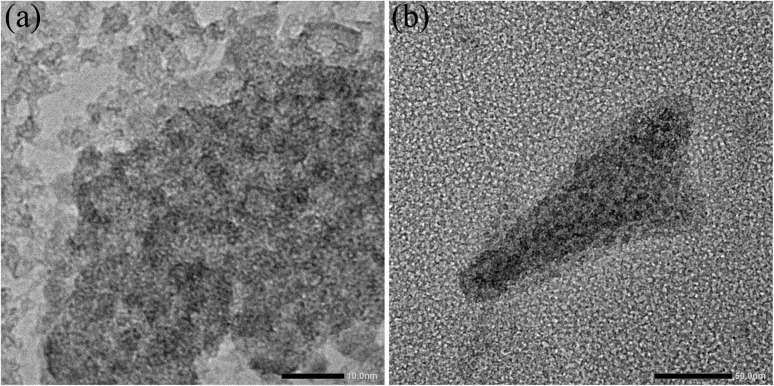
Transmission electron microscope (TEM) images of the iron (oxyhydr)oxide-based nanoparticles.

At 300 K, the Mössbauer spectra of both samples show a doublet with an isomer shift of 0.25 mm s^−1^ and an average electrical quadrupole splitting of 0.85 mm s^−1^. The doublet spectra is characteristic of the high spin of Fe^3+^.^24^ There is no evidence for the presence of Fe^2+^. This is in good agreement with the photometrically determined Fe^2+^ content of less than 1.5% of the total iron (see Tables S2–S9†). They are almost identical to the Mössbauer spectrum of the laboratory-scale drug sample.^24^ Likewise, the spectra recorded at 4.2 K compare well with the laboratory scale sample. Both samples from the 100 L syntheses show a magnetic sextet with an isomer shift of 0.24 mm s^−1^ and a mean electrical quadrupole splitting of −0.03 mm s^−1^, comparable to the laboratory scale reference sample.^24^ Only the mean hyperfine field of the pilot-scale samples of 44.0 and 44.4 T is slightly lower than that of the reference sample of 45.8 T. It is generally known that the hyperfine field at 4.2 K of iron (oxyhydr)oxides is reduced with decreasing particle size as the anisotropic energy of the particles is raised.^47^ Therefore, it is reasonable that the particles from the pilot-scale have slightly smaller crystal nuclei than those from the laboratory scale. These findings are in good agreement with the total particle size. Dynamic light scattering analyses reveal a significant reduction in particle size with increasing synthesis volume (see Tables S9 and Fig. S4†). In TEM images, it can be recognized that despite ultrasonic suspension, the ultra-small nanoparticles are not isolated, but are present in particle agglomerates consisting of individual small, dark, crystalline dots (iron (oxyhydr)oxide cores) in a lighter matrix (organic shells) (see Fig. 6a and b). However, the TEM images do not allow an accurate size estimation of the particle cores due to the agglomerates. Overall, the results of Mössbauer spectroscopy and DLS are consistent with the observation that particles from larger synthesis scales have a higher proportion of organic components. As discussed above, larger surface/volume ratios are expected with smaller crystal cores, wherefore more organic molecules can be bound to the ferrihydrite core. As a result, the total particle size is decreasing.

In summary, the FT-IR spectra show only minor changes in peak heights without new peaks, and the Mössbauer spectra are almost identical to those of the laboratory scale. Thus, ferrihydrite still seems to be the iron (oxyhydr)oxide structure in the core without any structure change due to the scale-up. However, there is evidence that the ferrihydrite core has decreased in size, presumably allowing more organic molecules to bind to the core surface. Therefore, the inulin and mannitol content in the particles has probably increased while the iron content has decreased. No significant changes are evident due to the scale-up for the essential properties, the yield, and the phosphate-binding efficacy. Overall, the results thus prove that these ultrasmall iron (oxyhydr)oxide nanoparticles can be successfully produced on a pilot scale.

## 4 Conclusions

This work aimed to demonstrate that ultrasmall iron (oxyhydr)oxide nanoparticles can be produced at a pilot scale-up to 100 L reaction volume. We have shown in the first part of this study that the synthesis described in this study is overall robust to changes in the synthesis conditions. Only changes in the synthesis parameters, such as temperature and stirring rate, lead to slight changes in the composition of the active substance. When different stirrer geometries were used, the composition remained unchanged and a comparably high phosphate binding efficiency and synthesis yield was achieved for all tested variations. In the subsequent stepwise scale-up from 176 mL to 100 L total volume, the composition of the active ingredient samples slightly changes in composition. As the synthesis scale increased, the inulin and mannitol content in the nanoparticles rose while the iron content decreased. FT-IR and Mössbauer spectroscopy indicated that the change in composition was presumably due to a reduction in the size of the crystal core, while the formation of other iron (oxyhydr)oxide structures could be ruled out. DLS analysis revealed that the total particle sizes also decrease with increasing synthesis volume. However, there was no change in the *in vitro* phosphate-binding efficacy and yield were evident.

Based on these results, the scale-up is considered to be successful. However, follow-up studies should be conducted to investigate in detail if the *in vivo* efficacy is not affected by the composition variation, especially regarding an increased inulin and mannitol content.

Overall, this successful scale-up demonstrated that ultrasmall iron (oxyhydr)oxide nanoparticles with almost constant properties and *in vitro* efficacy, as well as a yield > 40 g L^−1^, can be produced on a pilot scale by co-precipitation. These results support overcoming the obstacle of producing large quantities of nanoparticles by co-precipitation to exploit tailored iron (oxyhydr)oxide nanomaterials.

## Author contributions

All authors (except Friedrich Ernst Wagner, who deceased before the final version was finished) have read and agreed to the published version of the manuscript. Magdalena Teresa Spicher – conceptualization, methodology, formal analysis, investigation, writing—original draft preparation, visualization; Sebastian Patrick Schwaminger – conceptualization, writing—review and editing, supervision; Daniela von der Haar-Leistl – conceptualization, methodology, writing—review and editing, supervision, project administration; Marian Montiel Peralta – investigation. Writing—review and editing; Georgina Mikacevic – investigation, writing—review and editing; Friedrich Ernst Wagner – formal analysis, investigation, resources, writing—review and editing; Sonja Berensmeier – conceptualization, resources, writing—review and editing, supervision, project administration.

## Conflicts of interest

There are no conflicts to declare.

## Supplementary Material

RA-014-D4RA02719A-s001
