# Supplementary material for: Pilot-scale co-precipitation synthesis of a novel active ingredient made of ultrasmall iron (oxyhydr)oxide nanoparticles for the treatment of hyperphosphatemia
Source: RSC Adv. 2024 May 20;14(23):16117–27. doi: 10.1039/d4ra02719a (PMC11103348; doi:10.1039/d4ra02719a)
Supplement: RA-014-D4RA02719A-s001 [file RA-014-D4RA02719A-s001.pdf]

Electronic Supplementary Material (ESI) for RSC Advances.
This journal is © The Royal Society of Chemistry 2024

Electronic Supplementary Information (ESI)

Pilot-scale co-precipitation synthesis of ultrasmall iron (oxhydr)oxide nanoparticles

Magdalena Teresa Spicher,*[a,b] Sebastian Patrick Schwaminger,[b,c,d] Daniela von der Haar-Leistl,[a] Marian Montiel Parelta,[a] Georgina Mikacevic,[a] Friedrich Ernst Wagner,[e]‡ and Sonja Berensmeier[b]

Table S1 Synthesis conditions in the different scales

	total synthesis volume				
Synthesis parameter	176 mL	1.8 L	5.5 L	34.9 L	100 L
scaling factor (-)	1	10	31	198	568
stirring tank material	glass	glass	glass	stainless steel[1]	stainless steel[1]
volume solution 1 (L)	0.055	0.550	1.705	10.890	31.240
volume solution 2 (L)	0.114	1.140	3.534	22.572	64.752
hydrogen peroxide (L)	0.003	0.030	0.093	0.594	1.704

[1] V4A steel (tool number 1.4404)

Table S2 Impact of the synthesis temperature on the product yield Y, the phosphate-binding efficacy $q(PO_4^{3-})$ and the volumetric phosphate-binding capability $M(PO_4^{3-})$ at pH 3 and pH 7 as well as on the particle composition (the content of iron β(Fe), mannitol β(Man), inulin β(Inu), and gum Arabic β(GA)) as well as the proportion of divalent iron $c(Fe^{2+})$). The results are given as the mean value of at least three independent active ingredient samples prepared under the same conditions. Different letters indicate statistically significant differences (p-value < 0.05). When no letters are shown, no significant differences were identified

Characteristic	ANOVA p-value	Level 0: 4 °C		Level 1: 20 °C		Level 2: 40 °C	
Y (g/L)	0.25713	49.0 ± 3.3		47.8 ± 0.9		46.2 ± 1.8	
$q(PO_4^{3-})_{pH\ 3}$ (mg/g)	0.87298	200.9 ± 7.2		201.5 ± 5.1		203.7 ± 7.7	
$q(PO_4^{3-})_{pH\ 7}$ (mg/g)	0.67032[1]	115.5 ± 1.7		116.1 ± 3.9		113.9 ± 14.7	
$M(PO_4^{3-})_{pH\ 3}$ (mg/g)	0.73017	9.4 ± 0.1		9.6 ± 0.4		9.4 ± 0.6	
$M(PO_4^{3-})_{pH\ 7}$ (mg/g)	0.79368	5.4 ± 0.2		5.5 ± 0.3		5.3 ± 0.8	
β(Fe) (mg/g)	0.13352	218.6 ± 7.7		225.6 ± 6.5		228.1 ± 5.5	
β(Man) (mg/g)	0.42367[1]	126.9 ± 17.5		139.5 ± 2.2		133.9 ± 7.9	
β(Inu) (mg/g)	0.02253	183.0 ± 23.1	A	148.7 ± 15.5	B	149.1 ± 10.1	B
β(GA) (mg/g)	0.08237	349.0 ± 10.4		378.6 ± 18.3		371.4 ± 10.4	
$c(Fe^{2+})$ (%)	0.38733	1.1 ± 0.1		1.2 ± 0.1		1.3 ± 0.4	

[1] Use of the Kruskal-Wallis test due to variance heterogeneity

Table S3 Impact of the power input on the product yield Y, the phosphate-binding efficacy $q(PO_4^{3-})$ and the volumetric phosphate-binding capability $M(PO_4^{3-})$ at pH 3 and pH 7 as well as on the particle composition (the content of iron β(Fe), mannitol β(Man), inulin β(Inu), and gum Arabic β(GA)) as well as the proportion of divalent iron $c(Fe^{2+})$). The results are given as the mean value of at least three independent active ingredient samples prepared under the same conditions. Different letters indicate statistically significant differences (p-value < 0.05). When no letters are shown, no significant differences were identified

Characteristic	ANOVA p-value	Level -1: 0.03 kW/m³		Level 0: 0.16 kW/m³		Level 1: 0.93 kW/m³	
Y (g/L)	0.46597[1]	49.8 ± 0.8		49.0 ± 3.3		44.6 ± 5.3	
$q(PO_4^{3-})_{pH\,3}$ (mg/g)	0.06729	193.3 ± 4.4		200.9 ± 7.2		211.6 ± 10.1	
$q(PO_4^{3-})_{pH\,7}$ (mg/g)	0.50007	119.7 ± 6.5		115.5 ± 1.7		122.6 ± 10.3	
$M(PO_4^{3-})_{pH\,3}$ (mg/g)	0.4298[1]	9.6 ± 0.2		9.4 ± 0.1		9.5 ± 1.6	
$M(PO_4^{3-})_{pH\,7}$ (mg/g)	0.4298[1]	6.0 ± 0.4		5.4 ± 0.2		5.5 ± 1.1	
β((Fe) (mg/g)	0.01864	219.2 ± 1.6	B	218.6 ± 7.7	B	233.0 ± 2.6	A
β((Man) (mg/g)	0.05836	147.4 ± 7.7		126.9 ± 17.5		151.7 ± 4.4	
β((Inu) (mg/g)	0.02391[1]	156.5 ± 6.1	AB	183.0 ± 23.1	A	142.1 ± 5.7	B
β(GA) (mg/g)	0.37820	350.4 ± 6.0		349.0 ± 10.4		363.9 ± 19.7	
$c(Fe^{2+})$ (%)	0.59909	1.2 ± 0.1		1.1 ± 0.1		1.1 ± 0.1	

[1] Use of the Kruskal-Wallis test due to variance heterogeneity

Table S4 Impact of the titration time of the iron chloride solution on the product yield Y, the phosphate-binding efficacy $q(PO_4^{3-})$ and the volumetric phosphate-binding capability $M(PO_4^{3-})$ at pH 3 and pH 7 as well as on the particle composition (the content of iron β(Fe), mannitol β(Man), inulin β(Inu), and gum Arabic β(GA)) as well as the proportion of divalent iron $c(Fe^{2+})$). The results are given as the mean value of at least three independent active ingredient samples prepared under the same conditions. Different letters indicate statistically significant differences (p-value < 0.05). When no letters are shown, no significant differences were identified

Characteristic	ANOVA p-value	Level 0: 0 min		Level 1: 6 min		Level 2: 12 min	
Y (g/L)	0.54265	49.0 ± 3.3		47.6 ± 2.4		46.7 ± 2.6	
$q(PO_4^{3-})_{pH\,3}$ (mg/g)	0.49158	200.9 ± 7.2		212.1 ± 11.8		201.4 ± 19.4	
$q(PO_4^{3-})_{pH\,7}$ (mg/g)	0.68885 [1]	115.5 ± 1.7		125.6 ± 9.6		126.0 ± 21.4	
$M(PO_4^{3-})_{pH\,3}$ (mg/g)	0.19426	9.4 ± 0.1		10.1 ± 0.7		9.4 ± 0.4	
$M(PO_4^{3-})_{pH\,7}$ (mg/g)	0.44563	5.4 ± 0.2		6.0 ± 0.7		5.8 ± 0.6	
β((Fe) (mg/g)	0.59341	218.6 ± 7.7		229.3 ± 14.3		221.8 ± 25.1	
β((Man) (mg/g)	0.50549	126.9 ± 17.5		120.9 ± 14.5		134.8 ± 9.4	
β((Inu) (mg/g)	0.01530	183.0 ± 23.1	A	151.4 ± 25.3	AB	109.2 ± 36.5	B
β(GA) (mg/g)	0.20229	349.0 ± 10.4		351.8 ± 25.4		378.2 ± 17.8	
$c(Fe^{2+})$ (%)	0.36184	1.1 ± 0.1		1.1 ± 0.1		1.3 ± 0.3	

[1] Use of the Kruskal-Wallis test due to variance heterogeneity

Table S5 Impact of the co-precipitation time on the product yield Y, the phosphate-binding efficacy $q(PO_4^{3-})$ and the volumetric phosphate-binding capability $M(PO_4^{3-})$ at pH 3 and pH 7 as well as on the particle composition (the content of iron β(Fe), mannitol β(Man), inulin β(Inu), and gum Arabic β(GA)) as well as the proportion of divalent iron $c(Fe^{2+})$). The results are given as the mean value of at least three independent active ingredient samples prepared under the same conditions. Different letters indicate statistically significant differences (p-value < 0.05). When no letters are shown, no significant differences were identified

Characteristic	ANOVA p-value p-value	Level -1: 10 min		Level 0: 15 min		Level 1: 20 min		Level 2: 30 min	
Y (g/L)	0.17167	50.2 ± 4.0		49.0 ± 3.3		47.8 ± 2.7		44.6 ± 3.0	
$q(PO_4^{3-})_{pH\,3}$ (mg/g)	0.30887	188.9 ± 4.2		200.9 ± 7.2		194.0 ± 3.8		196.2 ± 10.2	
$q(PO_4^{3-})_{pH\,7}$ (mg/g)	0.17813	128.8 ± 0.3		115.5 ± 1.7		129.9 ± 8.0		135.3 ± 17.2	
$M(PO_4^{3-})_{pH\,3}$ (mg/g)	0.51960	9.5 ± 0.8		9.4 ± 0.1		9.3 ± 0.5		8.8 ± 0.9	
$M(PO_4^{3-})_{pH\,7}$ (mg/g)	0.29237	6.5 ± 0.5		5.4 ± 0.2		6.2 ± 0.2		6.1 ± 1.0	
β((Fe) (mg/g)	0.03653	221.5 ± 8.9	AB	218.6 ± 7.7	B	229.3 ± 10.8	AB	238.2 ± 9.0	A
β((Man) (mg/g)	0.09552*	127.3 ± 11.2		126.9 ± 17.5		127.0 ± 4.2		110.7 ± 6.0	
β((Inu) (mg/g)	0.18463	216.9 ± 27.4		183.0 ± 23.1		173.1 ± 25.9		194.0 ± 21.4	
β(GA) (mg/g)	0.04930*	316.3 ± 12.2	A	349.0 ± 10.4	A	333.4 ± 20.6	A	294.5 ± 25.7	A
$c(Fe^{2+})$ (%)	0.2259	1.2 ± 0.3		1.1 ± 0.1		1.4 ± 0.3		1.3 ± 0.2	

[1] Use of the Kruskal-Wallis test due to variance heterogeneity

Table S6 Impact of the oxidation time on the product yield Y, the phosphate-binding efficacy $q(PO_4^{3-})$ and the volumetric phosphate-binding capability $M(PO_4^{3-})$ at pH 3 and pH 7 as well as on the particle composition (the content of iron β(Fe), mannitol β(Man), inulin β(Inu), and gum Arabic β(GA)) as well as the proportion of divalent iron $c(Fe^{2+})$). The results are given as the mean value of at least three independent active ingredient samples prepared under the same conditions. Different letters indicate statistically significant differences (p-value < 0.05). When no letters are shown, no significant differences were identified

Characteristic	ANOVA p-value	Level -1: 10 min	Level 0: 15 min	Level 1: 20 min
Y (g/L)	0.20791	48.5 ± 0.9	49.0 ± 3.3	45.4 ± 2.8
$q(PO_4^{3-})_{pH\ 3}$ (mg/g)	0.14213	206.6 ± 15.8	200.9 ± 7.2	184.9 ± 11.5
$q(PO_4^{3-})_{pH\ 7}$ (mg/g)	0.22807	120.4 ± 10.2	115.5 ± 1.7	108.3 ± 9.1
$M(PO_4^{3-})_{pH\ 3}$ (mg/g)	0.05024[1]	10.0 ± 0.8	9.4 ± 0.1	8.4 ± 1.0
$M(PO_4^{3-})_{pH\ 7}$ (mg/g)	0.04235	5.8 ± 0.5	5.4 ± 0.2	4.9 ± 0.4
β((Fe) (mg/g)	0.43981	224.5 ± 10.8	218.6 ± 7.7	215.8 ± 7.9
β((Man) (mg/g)	0.16257	115.8 ± 9.1	126.9 ± 17.5	106.9 ± 7.2
β((Inu) (mg/g)	0.86421	183.7 ± 29.1	183.0 ± 23.1	191.7 ± 9.3
β(GA) (mg/g)	0.09567	315.7 ± 24.8	349.0 ± 10.4	337.1 ± 4.5
$c(Fe^{2+})$ (%)	0.12489	1.2 ± 0.3	1.1 ± 0.1	1.4 ± 0.1

[1] Use of the Kruskal-Wallis test due to variance heterogeneity

Table S7 Results of Pearson correlation test: the tested variables are the content of iron β(Fe), mannitol β(Man), inulin β(Inu), and gum Arabic β(GA), the product volumetric yield Y, the phosphate-binding efficacy $q(PO_4^{3-})$ at pH 7 and pH 3, and the volumetric phosphate-binding capability $M(PO_4^{3-})$ at pH 3 and pH 7. $M(PO_4^{3-})$ is derived from the phosphate-binding efficacy and the yield (see equation (5)), thus a correlation analysis between these factors is not meaningful. The p-value and the Pearson correlation coefficient r are provided for each correlation test. Significant correlations are assumed for p-value < 0.05

		β(Fe) (mg/g)						
$c(Fe^{2+})$ (%)	r<br>p-value	0.163<br>0.297	$c(Fe^{2+})$ (%)					
β(Man) (mg/g)	r<br>p-value	-0.056<br>0.721	-0.207<br>0.183	β(Man) (mg/g)				
β(Inu) (mg/g)	r<br>p-value	-0.117<br>0.457	0.091<br>0.562	-0.384<br>0.011	β(Inu) (mg/g)			
β(GA) (mg/g)	r<br>p-value	-0.137<br>0.406	-0.029<br>0.862	0.468<br>0.003	-0.757<br>< 0.01	β(GA) (mg/g)		
Y (g/L)	r<br>p-value	-0.439<br>0.003	-0.143<br>0.36	0.219<br>0.158	0.209<br>0.179	-0.056<br>0.733	Y (g/L)	
$q(PO_4^{3-})_{pH\ 3}$ (mg/g)	r<br>p-value	0.547<br>< 0.01	-0.033<br>0.844	0.142<br>0.39	-0.304<br>0.06	0.184<br>0.263	-0.004<br>0.982	$q(PO_4^{3-})_{pH\ 3}$ (mg/g)
$q(PO_4^{3-})_{pH\ 7}$ (mg/g)	r<br>p-value	0.671<br>< 0.01	0.029<br>0.862	-0.009<br>0.957	0.038<br>0.819	-0.312<br>0.053	0.053<br>0.748	0.296<br>0.067
$M(PO_4^{3-})_{pH\ 3}$ (g/L)	r<br>p-value	0.123<br>0.455	-0.126<br>0.444	0.294<br>0.069	-0.1<br>0.544	0.085<br>0.606	Not determined	Not determined
$M(PO_4^{3-})_{pH\ 7}$ (g/L)	r<br>p-value	0.356<br>0.026	-0.054<br>0.744	0.145<br>0.378	0.12<br>0.469	-0.292<br>0.071	Not determined	Not determined

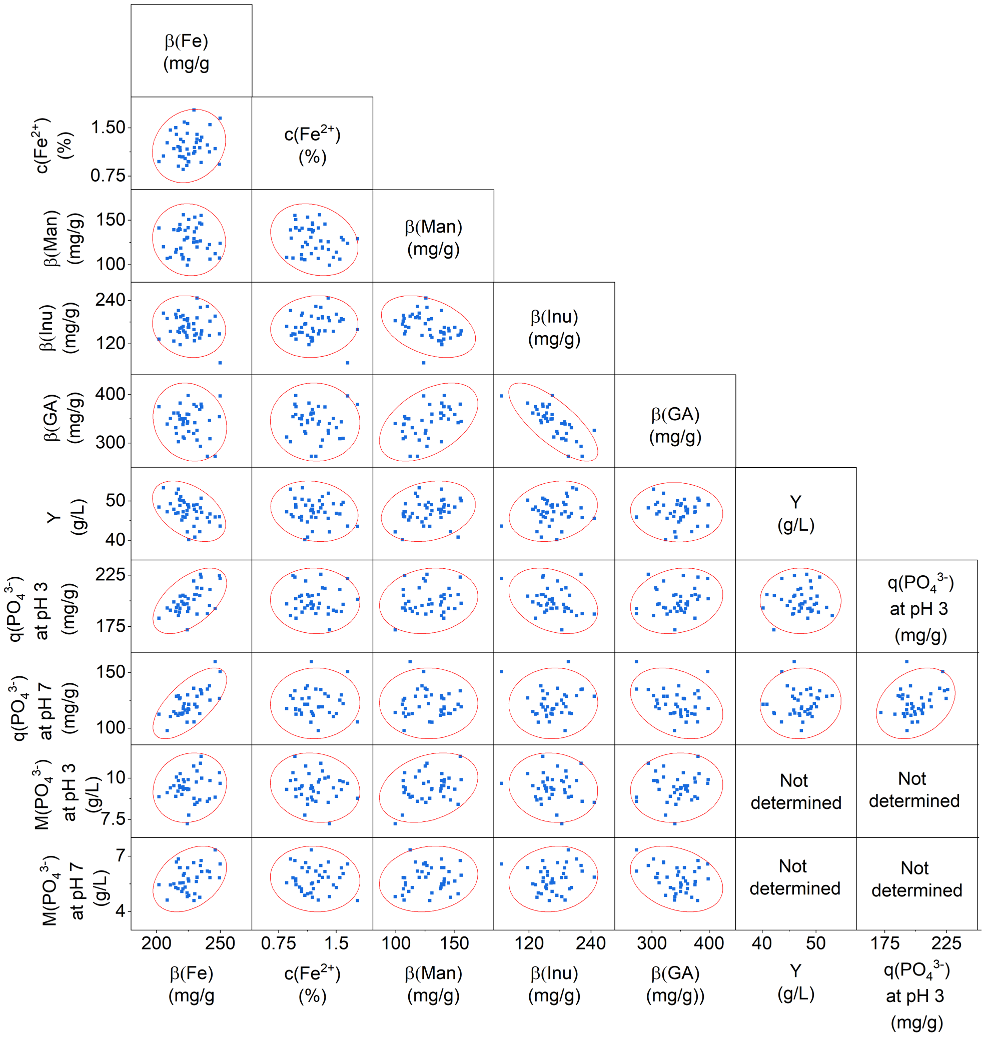

Fig. S1 Scatterplots and confidence ellipse for the correlation of the particle properties: the content of iron β(Fe), mannitol β(Man), inulin β(Inu), and gum Arabic β(GA), the product volumetric yield Y, the phosphate-binding efficacy q(PO_4^{3-}) at pH 7 and pH 3, and the volumetric phosphate-binding capability M(PO_4^{3-}) at pH 3 and pH 7. M(PO_4^{3-}) is derived from the phosphate-binding efficacy and the yield (see equation (5), thus a correlation analysis between these factors is not meaningful.

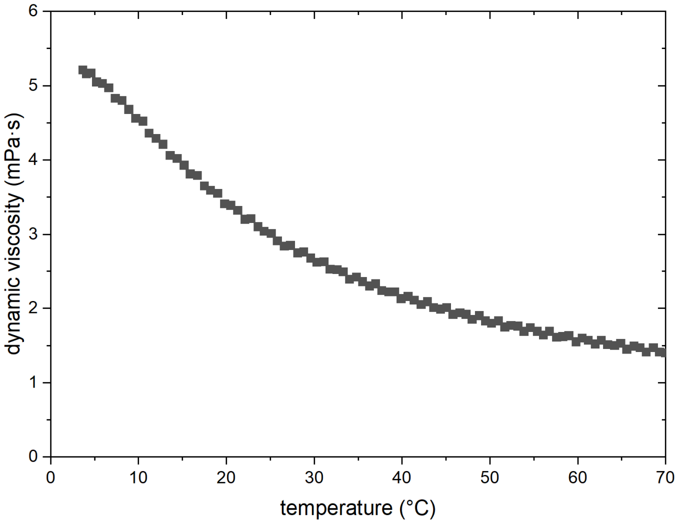

Fig. S2 Dynamic viscosity of the nanoparticle suspension with a dry matter concentration of 19 g/L in dependence on the temperature at a shear rate of 200/s.

Table S8 Results of the scale-up of the co-precipitation synthesis of the iron (oxyhydr)oxide nanoparticles: Impact of the synthesis scale on the product yield Y, the phosphate-binding efficacy $q(PO_4^{3-})$ and the volumetric phosphate-binding capability $M(PO_4^{3-})$ at pH 3 and pH 7 as well as on the particle composition (the content of iron β(Fe), mannitol β(Man), inulin β(Inu), gum Arabic β(GA), sodium β(Na), and chloride β(Cl)) as well as the proportion of divalent iron $c(Fe^{2+})$). The results are given as the mean value of at least three independent active ingredient samples prepared under the same conditions. Different letters indicate statistically significant differences (p-value < 0.05). When no letters are shown, no significant differences were identified

Characteristic	Kruskal-Wallis test p-value	Propeller stirrer (1-stage)	Propeller stirrer (2-stages)	Disk stirrer (1-stage)	Disk stirrer (2-stages)	Impeller stirrer	Pitched-blade stirrer	Counter-current stirrer
Y (g/L)	0.5402	44.0 ± 0.9	44.2 ± 1.2	38.0 ± 4.1	41.5 ± 5.9	41.3 ± 8.7	46.4 ± 3.4	45.4 ± 0.0
$q(PO_4^{3-})_{pH\,3}$ (mg/g)	0.1537	196.8 ± 9.1	206.5 ± 5.0	191.5 ± 2.2	199.3 ± 3.3	216.6 ± 8.0	212.6 ± 15.3	209.9 ± 6.8
$q(PO_4^{3-})_{pH\,7}$ (mg/g)	0.1399	106.0 ± 9.1	120.6 ± 2.7	101.9 ± 6.1	101.8 ± 6.1	107.6 ± 1.4	123.8 ± 9.2	108.3 ± 5.2
$M(PO_4^{3-})_{pH\,3}$ (mg/g)	0.2879	8.7 ± 0.2	9.1 ± 0.0	7.3 ± 0.9	8.3 ± 1.0	8.9 ± 1.6	9.9 ± 1.4	9.5 ± 0.3
$M(PO_4^{3-})_{pH\,7}$ (mg/g)	0.1399	4.7 ± 0.5	5.3 ± 0.3	3.9 ± 0.6	4.2 ± 0.9	4.4 ± 0.9	5.8 ± 0.8	4.9 ± 0.2
β(Fe) (mg/g)	0.2299	216.3 ± 10.6	213.7 ± 5.7	227.1 ± 9.8	225.5 ± 7.5	230.6 ± 2.0	227.4 ± 12.3	240.6 ± 7.9
β(Man) (mg/g)	0.0911	139.1 ± 16.7	146.8 ± 0.3	107.8 ± 17.3	117.1 ± 5.0	131.9 ± 13.1	133.4 ± 11.7	115.3 ± 5.1
β(Inu) (mg/g)	0.2258	166.2 ± 7.1	183.9 ± 12.3	152.4 ± 15.3	136.0 ± 2.2	154.9 ± 28.6	140.1 ± 5.1	142.5 ± 12.1
β(GA) (mg/g)	0.2218	355.8 ± 2.9	285.9 ± 2.7	325.1 ± 3.3	328.7 ± 4.3	283.8 ± 4.0	295.1 ± 48.6	341.0 ± 27.4
$c(Fe^{2+})$ (%)	0.1596	1.1 ± 0.0	1.1 ± 0.0	0.7 ± 0.0	1.0 ± 0.0	1.1 ± 0.4	0.9 ± 0.2	0.8 ± 0.1

Table S9 Results of the scale-up of the co-precipitation synthesis of the iron (oxyhydr)oxide nanoparticles: Impact of the synthesis scale on the product yield Y, the phosphate-binding efficacy $q(PO_4^{3-})$ and the volumetric phosphate-binding capability $M(PO_4^{3-})$ at pH 3 and pH 7, the mean particle size d_{50} as well as on the particle composition (the content of iron β(Fe), mannitol β(Man), inulin β(Inu), gum Arabic β(GA), sodium β(Na), and chloride β(Cl)) as well as the proportion of divalent iron $c(Fe^{2+})$). The results are given as the mean value of at least three independent active ingredient samples prepared under the same conditions. Different letters indicate statistically significant differences (p-value < 0.05). When no letters are shown, no significant differences were identified

Characteristic	ANOVA p-value	0.2 L		1.8 L		5.5. L		34.9 L		100 L	
Y (g/L)	0.30844[1]	45.0 ± 3.9		39.7 ± 0.6		37.2 ± 6.6		49.5 ± 3.3		46.9 ± 10.1	
$q(PO_4^{3-})_{pH\,3}$ (mg/g)	0.39436	198.2 ± 7.4		216.6 ± 8.0		189.8 ± 26.4		183.1 ± 10.0		199.7 ± 3.5	
$q(PO_4^{3-})_{pH\,7}$ (mg/g)	0.44012	119.2 ± 8.0		107.6 ± 1.4		137.2 ± 29.2		113.7 ± 0.8		126.2 ± 2.2	
$M(PO_4^{3-})_{pH\,3}$ (mg/g)	0.34579[1]	8.9 ± 0.4		8.6 ± 0.2		6.5 ± 0.4		9.1 ± 1.1		9.4 ± 2.2	
$M(PO_4^{3-})_{pH\,7}$ (mg/g)	0.60445[1]	5.4 ± 0.1		4.3 ± 0.0		5.4 ± 2.4		5.6 ± 0.3		5.9 ± 1.4	
β(Fe) (mg/g)	0.02408	216.4 ± 4.2	AB	230.6 ± 2.0	A	203.5 ± 15.4	AB	185.6 ± 16.6	B	194.2 ± 6.3	AB
β(Man) (mg/g)	0.00203	119.7 ± 9.5	A	122.5 ± 7.0	A	137.3 ± 10.2	AB	159.9 ± 4.0	B	162.4 ± 3.6	B
β(Inu) (mg/g)	0.00867	136.0 ± 7.6	A	141.4 ± 18.5	A	185.6 ± 23.4	AB	178.5 ± 23.1	AB	227.7 ± 24.0	B
β(GA) (mg/g)	0.13254[1]	302.7 ± 28.9		283.8 ± 4.0		287.4 ± 4.3		250.3 ± 40.5		220.9 ± 11.5	
β(Na) (mg/g)	0.10759	9.1 ± 0.4		7.7 ± 0.0		10.0 ± 1.3		9.4 ± 1.4		10.7 ± 0.7	
β(Cl) (mg/g)	0.99316[1]	0.6 ± 0.1		0.8 ± 0.4		0.7 ± 0.3		0.6 ± 0.1		0.6 ± 0.1	
$c(Fe^{2+})$ (%)	0.59272[1]	0.8 ± 0.1		1.1 ± 0.4		0.8 ± 0.1		0.9 ± 0.1		0.8 ± 0.1	
d_{50} (nm)	0.01391	80.3 ± 17.6	B	48.5 ± 16.9	AB	45.7 ± 13.3	AB	31.2 ± 0.2	A	22.5 ± 1.7	A

[1] Use of the Kruskal-Wallis test due to variance heterogeneity

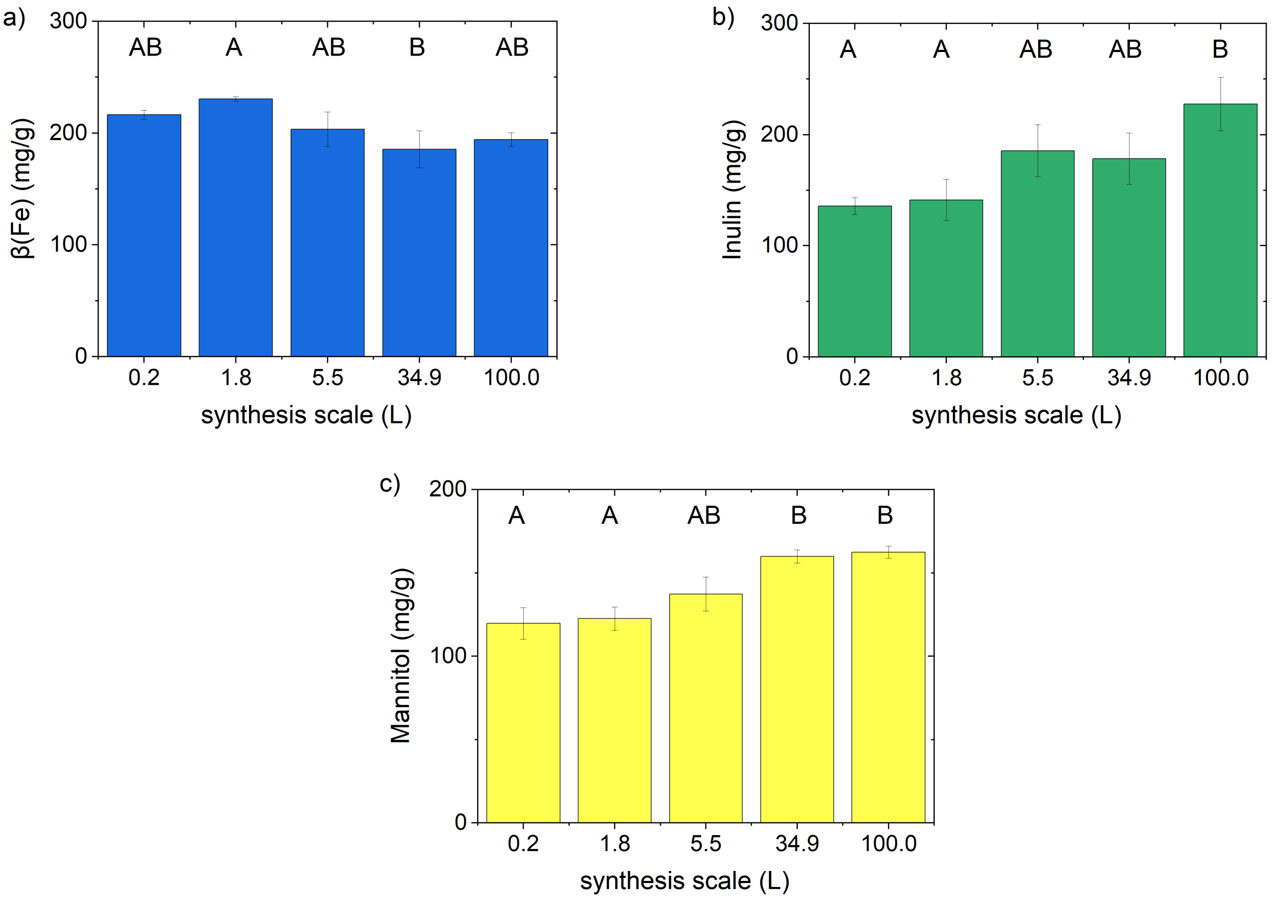

Fig. S3 Composition of the active ingredient synthesized in different scales: a) iron content $\beta(\mathrm{Fe})$, b) inulin content $\beta(\mathrm{Inu})$ and c) mannitol content $\beta(\mathrm{Man})$. The results are given as the mean value of at least two independent active ingredient samples prepared under the same conditions. Different letters indicate statistically significant differences (p-value < 0.05).

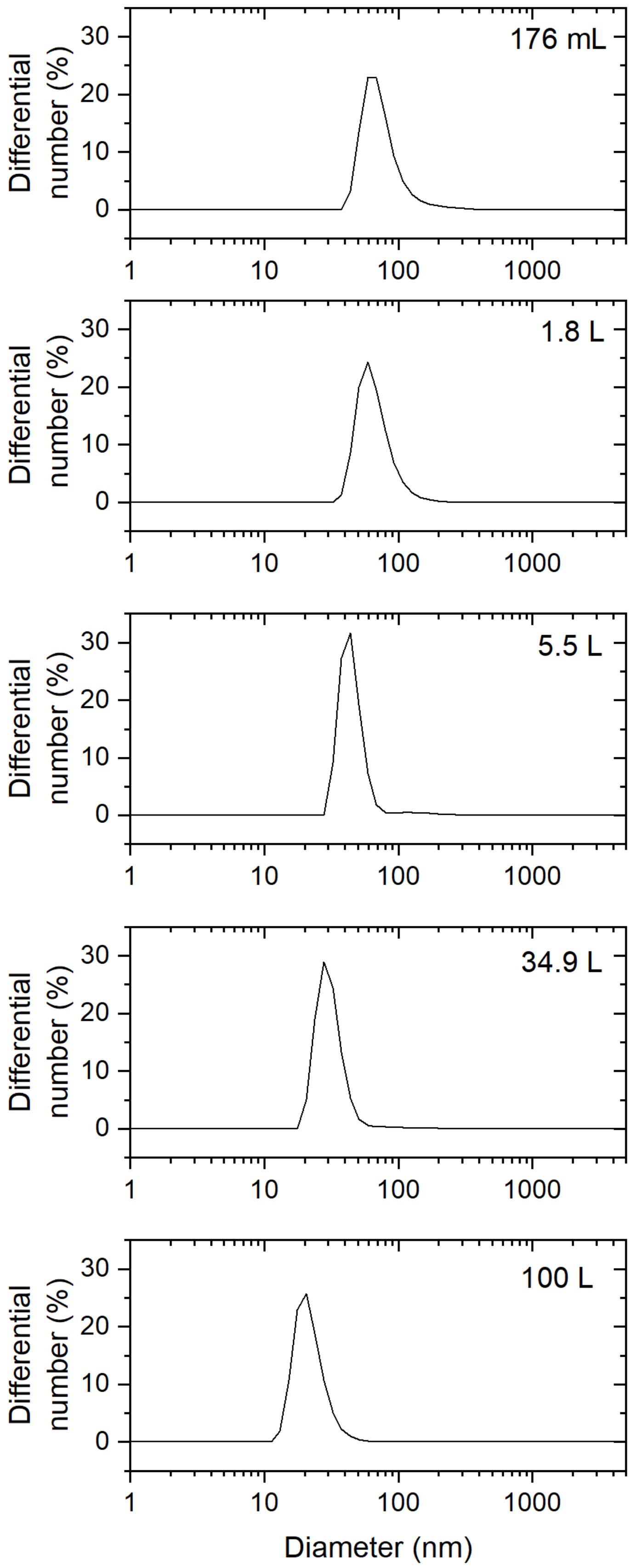

Fig. S4 Particle size distribution measured by dynamic light scattering (DLS) of active ingredient samples synthesized in different scales.
